# Altered adult brain morphology in a mouse model of late-onset fetal growth restriction

**DOI:** 10.1162/IMAG.a.946

**Published:** 2025-10-21

**Authors:** Ruiyan Tan, Lindsay S. Cahill, Shoshana Spring, Anastasia Smolina, Anum Rahman, Darren Fernandes, Mike Seed, Christopher K. Macgowan, John C. Kingdom, John G. Sled

**Affiliations:** Department of Medical Biophysics, University of Toronto, Toronto, ON, Canada; Mouse Imaging Centre, The Hospital for Sick Children, Toronto, ON, Canada; Translational Medicine, SickKids Research Institute, Toronto, ON, Canada; Department of Chemistry, Memorial University of Newfoundland, St. John’s, NL, Canada; Department of Obstetrics & Gynecology, University of Toronto, Toronto, ON, Canada; Maternal-Fetal Medicine Division, Department of Obstetrics & Gynaecology, Mount Sinai Hospital, Toronto, ON, Canada

**Keywords:** longitudinal imaging, MRI, postnatal brain development, late-onset fetal growth restriction, mouse model, developmental origins of health and disease

## Abstract

Late-onset fetal growth restriction (FGR) from placental insufficiency results in progressive fetal hypoxia and is often associated with poor neurodevelopmental outcomes. In response to hypoxia, fetal brain sparing physiology is activated to protect the developing brain from injury via enhancing oxygen delivery. However, this protection may not be robust, and some brain regions remain susceptible to hypoxic injury. In addition, not all injury is manifested during fetal life, and may present in infant development. Using a mouse model of late-onset FGR where gestation is extended for 24 h, equivalent to approximately 2 weeks in humans, we deployed longitudinal *in vivo* magnetic resonance imaging to assess structural brain development from birth to adulthood. Postterm fetuses showed significantly smaller volumes of the perirhinal and ectorhinal cortex regions compared to controls, with no differences observed between sexes. These brain regions, essential for recognition memory, exhibited progressively greater volume reductions with increasing postnatal age. Our findings add to the growing body of literature demonstrating that an adverse fetal environment during critical periods of development can have effects on the brain that persist into adulthood.

## 1 Introduction

Fetal hypoxia driven by placental insufficiency is a common pregnancy complication associated with abnormal neurodevelopment (S. L. Miller et al., 2016) and hypoxic-ischemic brain injury (Jensen et al., 1999). Placental insufficiency describes conditions in which the placenta can no longer sustain the energy demands of the growing fetus, resulting in insufficient oxygen and nutrient delivery to the fetus (S. L. Miller et al., 2016). Placental insufficiency can result from a variety of pregnancy complications, including the prolongation of normal gestational term. Past term, the fetus outgrows the functional capacity of the placenta, resulting in fetal hypoxia and growth restriction (Rahman et al., 2017; Vorherr, 1975).

In response to hypoxia, a fetal compensatory mechanism called brain sparing is initiated, which maintains oxygen to the brain by increasing blood flow at the expense of other organs such as the liver (J. Miller et al., 2008; Pearce, 2006). Both murine and human fetuses have been shown to brain spare under acute and chronic hypoxic conditions (Cahill et al., 2014, 2019; Giussani, 2016). This adaptation is sex dependent, with males more susceptible to hypoxic insults than females (Hill & Fitch, 2012; Rosenfeld, 2015). While brain sparing is a known indicator of fetal distress, the postnatal neurodevelopmental consequences of this mechanism are variable and poorly understood. Studies using mouse models of fetal hypoxia (Cahill et al., 2019) and human growth-restricted fetuses (Yanney & Marlow, 2004) have shown normal brain development. Yet, other human studies indicate that brain sparing does not completely protect against neurodevelopmental injury (Benítez-Marín et al., 2021).

Our group has described a mouse model of late-onset fetal growth restriction (FGR), where prolongation of regular gestational term via maternal progesterone injections resulted in fetal hypoxia, hypoxic fetal brain injury, and eventual fetal demise (Rahman et al., 2017). Despite an increase in umbilical artery blood flow from embryonic day 18.5 (E18.5, term) to E19.5 (1 day postterm), the placental weight decreased significantly and the fetoplacental arterial vasculature did not continue to remodel, indicating that the postterm murine placenta is unable to safely sustain the fetus. A recent follow-up study by our group found these postterm fetuses brain spared and showed that a weaker brain-sparing response (less redistribution of blood flow to the fetal brain) was associated with cerebral hypoxia and stillbirth (Smolina et al., 2023). The fetal cerebral hypoxia generated by this mouse model of late-onset FGR, therefore, provides an opportunity to study how these processes affect postnatal brain development.

Longitudinal *in vivo* magnetic resonance imaging (MRI) is a powerful method to study the whole mouse brain in the same animal over time (Henkelman, 2010). Mice can be imaged as early as postnatal day 1 (Szulc et al., 2015), and systemic administration of manganese chloride provides excellent contrast to delineate brain structures Here, we use our established mouse model of late-onset FGR and longitudinal *in vivo* MRI to investigate the impact of fetal hypoxia on neuroanatomy from early life until adulthood in both male and female offspring.

## 2 Methods

### 2.1 Study summary

**Fig. 1.**
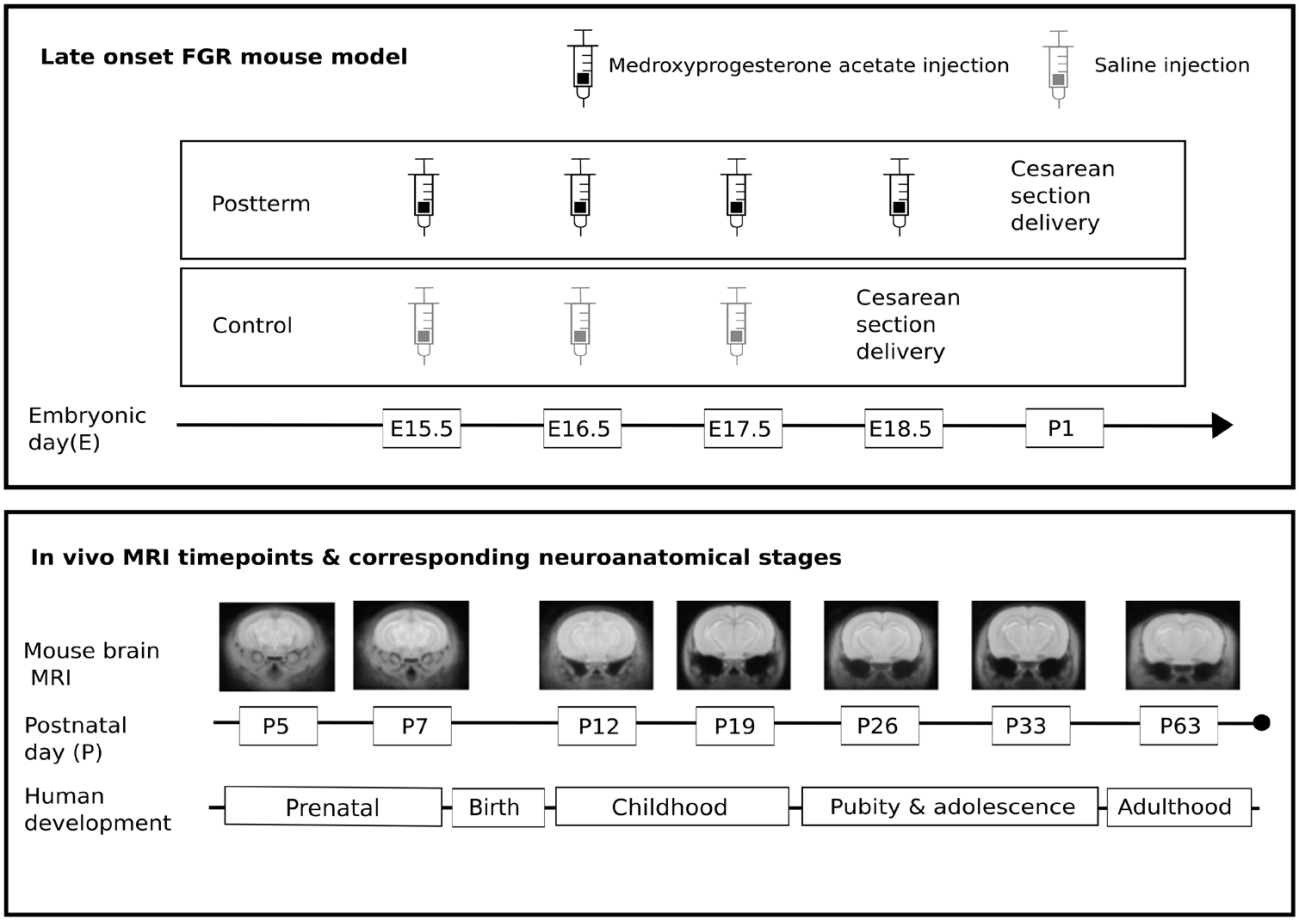
Timeline of experimental procedures. A mouse model of late-onset FGR using prenatal maternal injections of medroxyprogesterone acetate, extended pregnancy by 24 h. Controls received saline injections. All pups were delivered by Cesarean section. Postnatally, pups were scanned using *in vivo* MRI to assess neuroanatomy at various developmental periods corresponding to important human neuroanatomical stages. Fetal growth restriction: FGR, Magnetic resonance imaging: MRI.

The objective of the present study was to investigate how antenatal hypoxia affects postnatal neurodevelopment using a mouse model. Using injections of exogenous progesterone, we delayed labor by 24 h, which, when scaled proportionally to total gestation, is equivalent to approximately 2 weeks in human pregnancy. Fetuses were delivered by Cesarean section at standard term age for the control group (full term is 18.5 in CD-1 mice) and 1 day postterm for the postterm group. All pups were fostered to a CD-1 dam with an age-matched litter. To normalize rearing and ensure equal manganese intake by the pups through the maternal milk, the litter size was reduced to 6 for each dam. The mice were scanned longitudinally using *in vivo* manganese-enhanced magnetic resonance imaging (MEMRI) at 7 postnatal (P) time points, days P5, P7, P12, P19, P26, P33, and P63 to assess neuroanatomy at major developmental milestones (Fig. 1). Four pups from each dam (where possible, two male and two female) were used for longitudinal scanning. Mice were weaned at P28 and separated into cages based on sex.

### 2.2 Animals

Experimental procedures were approved by the Animal Care Committee at The Centre for Phenogenomics and conducted in accordance with the guidelines established by the Canadian Council on Animal Care. Fourteen adult female CD-1 mice (6–12 weeks of age) were purchased from Charles River Laboratories (St. Constant, QC, Canada) and mated in-house. The morning that a vaginal copulation plug was detected was designated as embryonic day (E) 0.5. We considered term age to be P0 and, therefore, the postterm group was born at P1. With this naming, we are correcting for the additional day *in utero* so that the MRI scans are completed on the same day post-conception.

### 2.3 Progesterone administration

From E15.5 to E18.5, pregnant mice were randomized to either a postterm pregnancy group which received a daily subcutaneous injection of 16 mg/kg medroxyprogesterone acetate (Sigma-Aldrich, St. Louis, MO, USA) in 0.4 mL sterile saline (Shynlova et al., 2008), or a control group that received 0.4 mL of sterile saline (Fig. 1).

### 2.4 Cesarean section and fostering

The dams were sacrificed by cervical dislocation, and a Cesarean section was performed as previously described (Murphy, 1993). Briefly, the uterus was exposed through a vertical lower abdominal incision (~1 cm) and removed at the cervical end. The pups were removed from the yolk sac and amnion using blunt forceps, the membranes dissected, and the umbilical cord cut. Birth weights of pups and placentas were collected. The pups were kept warm and transferred to a CD-1 dam that was removed from her age-matched litter. Three fetuses from the left uterine horn and three fetuses from the right uterine horn were chosen for fostering. Three of the groups had to be excluded because the foster dams showed signs of stress and cannibalized some or all the pups during the first 24–48 h.

### 2.5 In vivo magnetic resonance imaging

Twenty-four hours prior to each imaging session, mice were injected intraperitoneally with the contrast agent, manganese chloride (MnCl_2_) (0.4 mmol/kg) (Wadghiri et al., 2004). For mice 10 days and younger, MnCl_2_ is received through the maternal milk via an intraperitoneal injection to the dam. Neuroanatomical images were acquired using a multi-channel 7.0 Tesla, 30 cm diameter bore magnet MRI scanner (Bruker, Billerica, Massachusetts) that allowed 4 mice to be simultaneously imaged using cryogenically cooled surface coils (Arbabi et al., 2022). The mice were kept warm using a heated water platform. The mice were anaesthetized with 1–2% isoflurane in 100% oxygen, and respiration was monitored throughout the scan using self-gated signals (Nieman et al., 2009). The imaging protocol consisted of a T1-weighted FLASH 3D gradient echo sequence with the following parameters: TR = 26 ms, TE = 8.25 ms, flip angle = 23°, field-of-view = 25 x 22 x 22 mm, matrix = 334 x 294 x 294, 2 averages, and 75 μm isotropic image resolution. Following image acquisition, MR images were corrected for gradient field inhomogeneity-induced distortions and intensity non-uniformity (Nieman et al., 2018; Sled et al., 1998).

### 2.6 Longitudinal image registration and volume analysis

Images were registered using an adapted pipeline in the PydPiper toolkit to account for changes across time (Friedel et al., 2014; Qiu et al., 2018). Briefly, images were first registered to one another within each time point, resulting in a consensus average for each age. Then, each consensus image was registered to the following time point for all time points. Structural brain volume changes between the groups were determined using the MAGeT Brain algorithm (Chakravarty et al., 2012) with an input atlas containing 182 brain structures (Dorr et al., 2008; Qiu et al., 2018; Richards et al., 2011; Steadman et al., 2014; Ullmann et al., 2013). Within each timepoint, individual images were quality checked for correct orientation and adequate contrast and resolution. Images that did not meet these requirements were removed before running MAGeT. The MAGeT outputs were quality controlled by visualizing the atlas overlaid on top of the brain image and ensuring all areas were covered with the atlas. The 7 atlases were defined at P5, 7,10,17, 29, 36, 65 dates, which approximated this study’s scan dates (P5, 7, 12, 19, 26, 33, 63) to 0–3 days.

### 2.7 Statistical analysis

All statistical tests were conducted using the R statistical software (version 4.2.3, http://www.r-project.org). Fetal weight, placental weight, and fetal placental weight ratio (FPWR) were analyzed using a linear mixed-effects model to assess the effect of the group while accounting for litter as a random effect. Fetal placental weight ratios were calculated by dividing birth weights by placental weights. Similarly, maternal weight was modeled with a linear mixed-effects model with group and gestational age as fixed effects and dam as random effect, allowing for interactions. To assess if the rate of maternal weight postterm growth deviated from normal gestational term, a linear mixed-effects model was fit with age and gestational interval as fixed effects and dam as random effect allowing interactions. Body weight was analyzed using a linear mixed-effects model with group, age, and sex as fixed effects, allowing interactions between age and sex, and accounting for litter as a random effect.

Neuroanatomical data were analyzed using linear mixed-effects models, first for whole brain volumes and then independently for each structure. Fixed effects included a shared non-linear growth pattern of age (modeled with a cubic natural spline), group, sex, and group-sex interactions and a separate linear age term describing the difference between groups as a function of age. A random effect was included to account for inter-mouse variability. Neuroanatomical data were organized into a hierarchy defined by the Allen Brain Atlas (Sunkin et al., 2013), and the same linear mixed-effects model was used for hierarchical analysis (Supplementary Methods). Relative brain volumes were calculated by dividing structural volume by whole-brain volume and multiplying by 100 to get a percentage of whole brain volume. Voxel-wise analysis was performed by fitting each voxel with the model above. The results of voxel-wise analysis replicated the structure-wise results, hence only structure-wise analysis is presented. For all fitted models, AIC was used to select the model that adequately described the data.

An ANOVA was performed to determine whether any of the following variables affected weights and brain volumes. For weights, the variables considered were group, age, sex and age by sex interactions. For brain volumes, the variables considered were the 3 age-dependent spline terms, group, sex, group by sex interactions, and the linear age term. Degrees of freedom were approximated using Satterthwaite’s method (Satterthwaite, 1946). The reported p-values were corrected for multiple comparisons using the false discovery rate (FDR) method (Genovese et al., 2002). Statistical significance was determined at an FDR threshold of 10%, and p-values were reported unadjusted. All figures show the fitted model and 95% confidence intervals calculated based on the estimated covariance of the fixed effects.

## 3 Results

Total number of MRI scans per experimental group used in the statistical analysis are shown in Table 1. The study cohort comprised 11 litters and 43 pups. One postterm litter (4 pups) met the criteria for humane endpoint (poor weight gain) and was excluded from analysis. An additional 2 pups (1 control and 1 postterm) were excluded due to death before P12. In total, there were 10 litters and 37 pups (18 postterm pups, 19 control pups) that were viable from P5. The lower scan numbers compared to group numbers is due to a total of 7 deaths during the scan caused by equipment malfunction and/or excluded due to poor image quality from motion.

**Table 1.** Total MRI scans per experimental group used in analysis.

	P5	P7	P12	P19	P26	P33	P63
Control	14 (9 M, 5F)	17 (8 M, 9F)	16 (7 M, 9F)	17 (8 M, 9F)	15 (7 M, 8F)	15 (7 M, 8F)	13 (5 M, 8F)
Postterm	18 (11 M, 7F)	17 (10 M, 7F)	14 (7 M, 7F)	17 (10 M, 7F)	18 (11 M, 7F)	14 (8 M, 6F)	15 (9 M, 6F)

MRI scan numbers for each group after removing poor quality images per age in each experimental group.

Magnetic resonance imaging: MRI, Postnatal day: P, Female: F, Male: M.

**Fig. 2.**
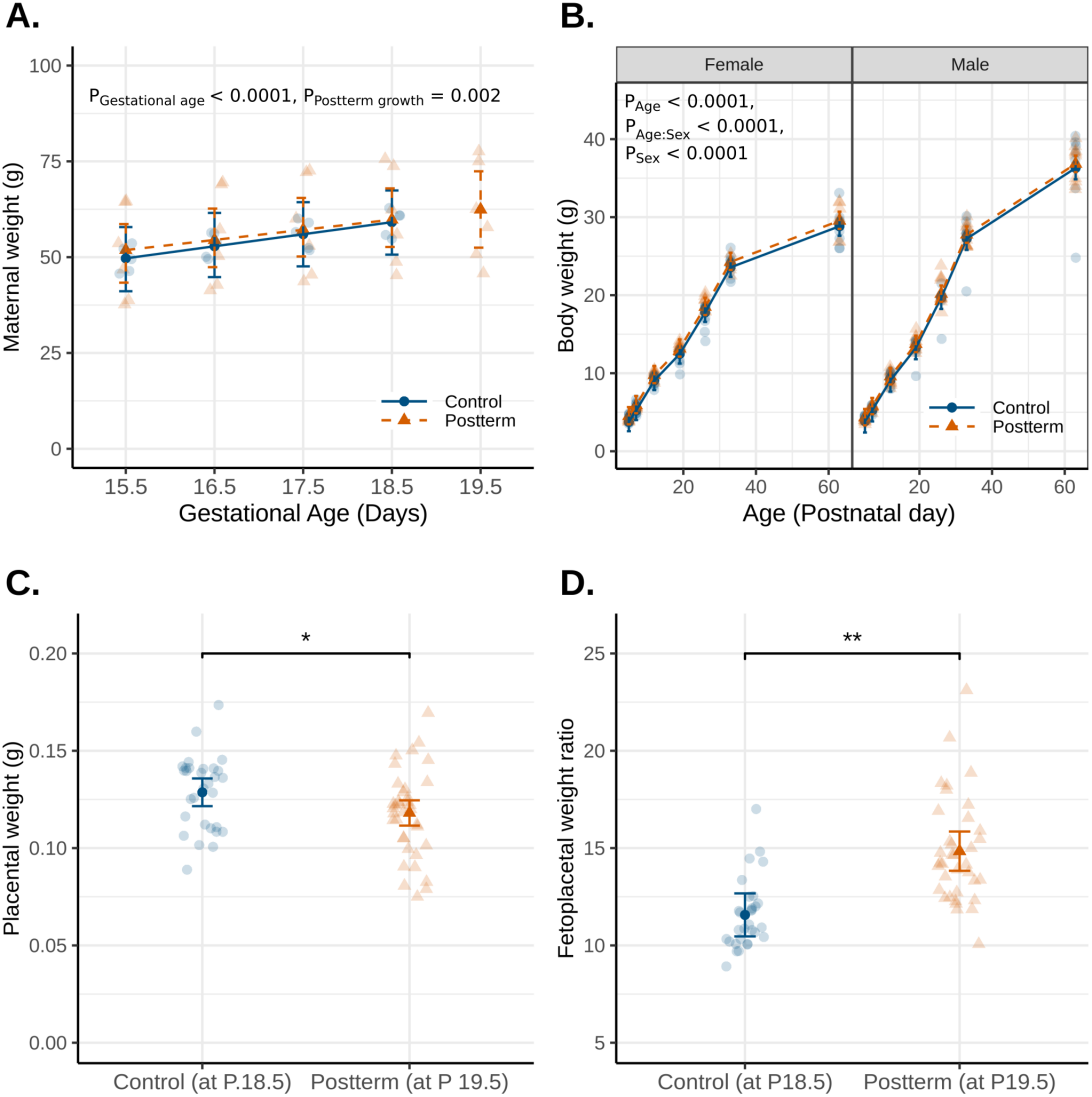
(A) Maternal weights were recorded during pregnancy from E15.5-birth (n = 5 dams/group). (B) Postnatal body weight of pups was recorded from birth to adulthood. (C) Placental weights were recorded at birth along with fetal weights to calculate the (D) fetoplacental weight ratio. *p < 0.05, **p < 0.005. Main effects of gestational age, postterm growth, age and sex are denoted as p_Gestational age_, p_Postterm growth_, p_Age_ and p_Sex_. The sex-by-age interaction is denoted by p_Age:Sex._

Maternal weight significantly increased with gestational age (p < 0.0001) (Fig. 2A). The maternal weight at the postterm age significantly differed from the linear growth trajectory estimated from the normal gestational term weights (p = 0.002) (Fig. 2A). Postnatally, body weights of offspring showed significant effects of age (p < 0.0001), sex (p < 0.0001) with a sex-by-age interaction (p < 0.0001) (Fig. 2B). Placental weights were decreased in the postterm group compared to controls (p = 0.03, Fig. 2C) with fetoplacental weight ratios (FPWR) significantly higher in the postterm group compared to controls (p = 0.002, Fig. 2D). A high FPWR is indicative of an imbalance between the placenta’s capacity to support a growing fetus and the fetus’s size (Perry et al., 1995).

**Fig. 3.**
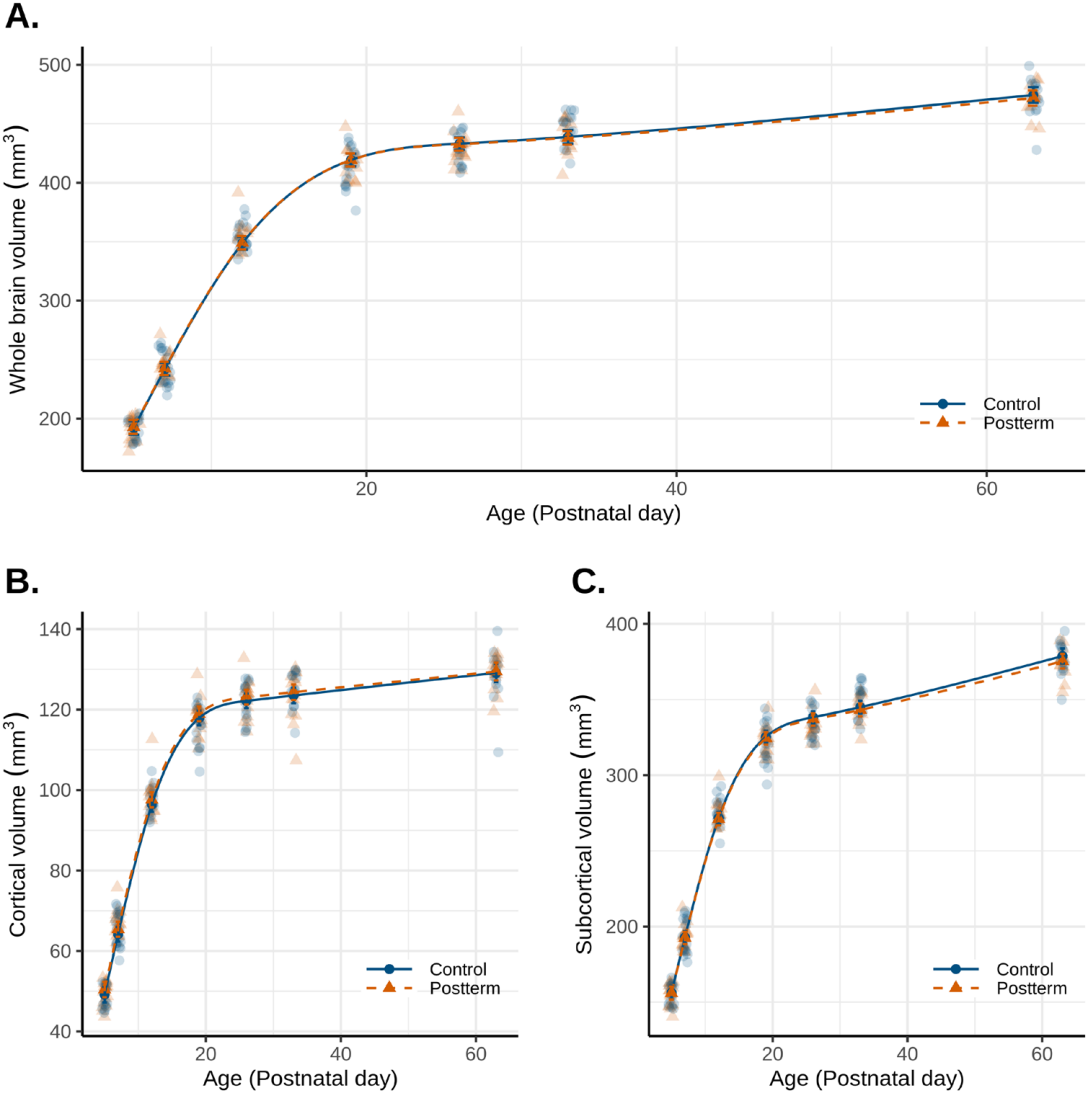
Whole brain and cortical area volume growth. Absolute volumes of (A) the whole brain, (B) cortical areas, and (C) subcortical areas over time measured using MRI. n = 13–18 per sex from 5 litters per group.

Whole-brain volumes did not significantly differ between postterm and control (Fig. 3A). Growth patterns of cortical areas (cortical areas included: piriform cortex, entorhinal cortex, frontal cortex, parietal cortex, cingulate cortex, temporal cortex, insular cortex including perirhinal and ectorhinal cortex, and occipital cortex) (Fig. 3B) and subcortical areas (Fig. 3C) were also similar between the groups.

**Fig. 4.**
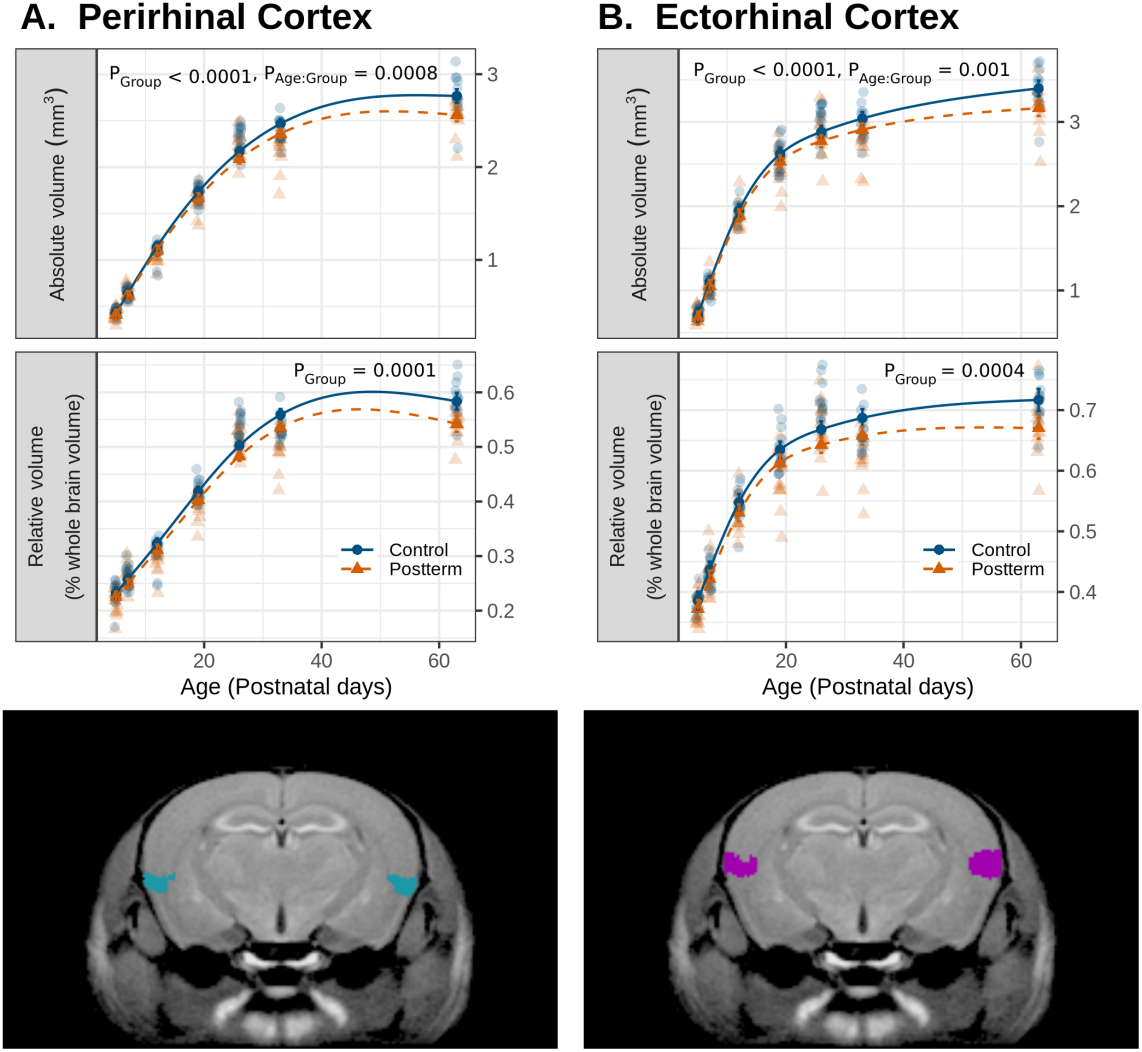
Structural volume changes. Absolute and relative to whole brain volumes of the (A) perirhinal cortex and (B) ectorhinal cortex growth trajectories over several time points measured using MRI. The structure segmentation is overlaid in colour (perirhinal cortex in blue and ectorhinal cortex in purple) on a coronal slice of the consensus MR brain.

While gross brain morphology was similar between groups, the postterm pregnancy group did show altered morphology of certain brain structures. Overall, there was a significant main effect of group for the perirhinal cortex, both in absolute volume (p < 0.0001) and relative to whole-brain volumes (p = 0.0001) (Fig. 4A). There was also a significant main effect of group in the absolute (p < 0.0001) and relative volumes (p = 0.0004) of the ectorhinal cortex (Fig. 4B). For both the perirhinal and ectorhinal cortex absolute volumes, there was a significant age by group interaction (p = 0.0008 for perirhinal cortex, p = 0.001 for ectorhinal cortex). The largest absolute volume differences between the postterm and control groups were at P63 with 7.00% smaller perirhinal cortices and 6.3% smaller ectorhinal cortices in the postterm group compared to controls. Sex differences were present in relative volumes of the medial amygdala volumes (p = 0.0005) (Supplementary Fig. 1A). Relative volumes of the copula pyramis lobule, a cerebellar grey matter structure, were significantly larger in the postterm group compared to control (p = 0.001) (Supplementary Fig. 1B).

**Fig. 5.**
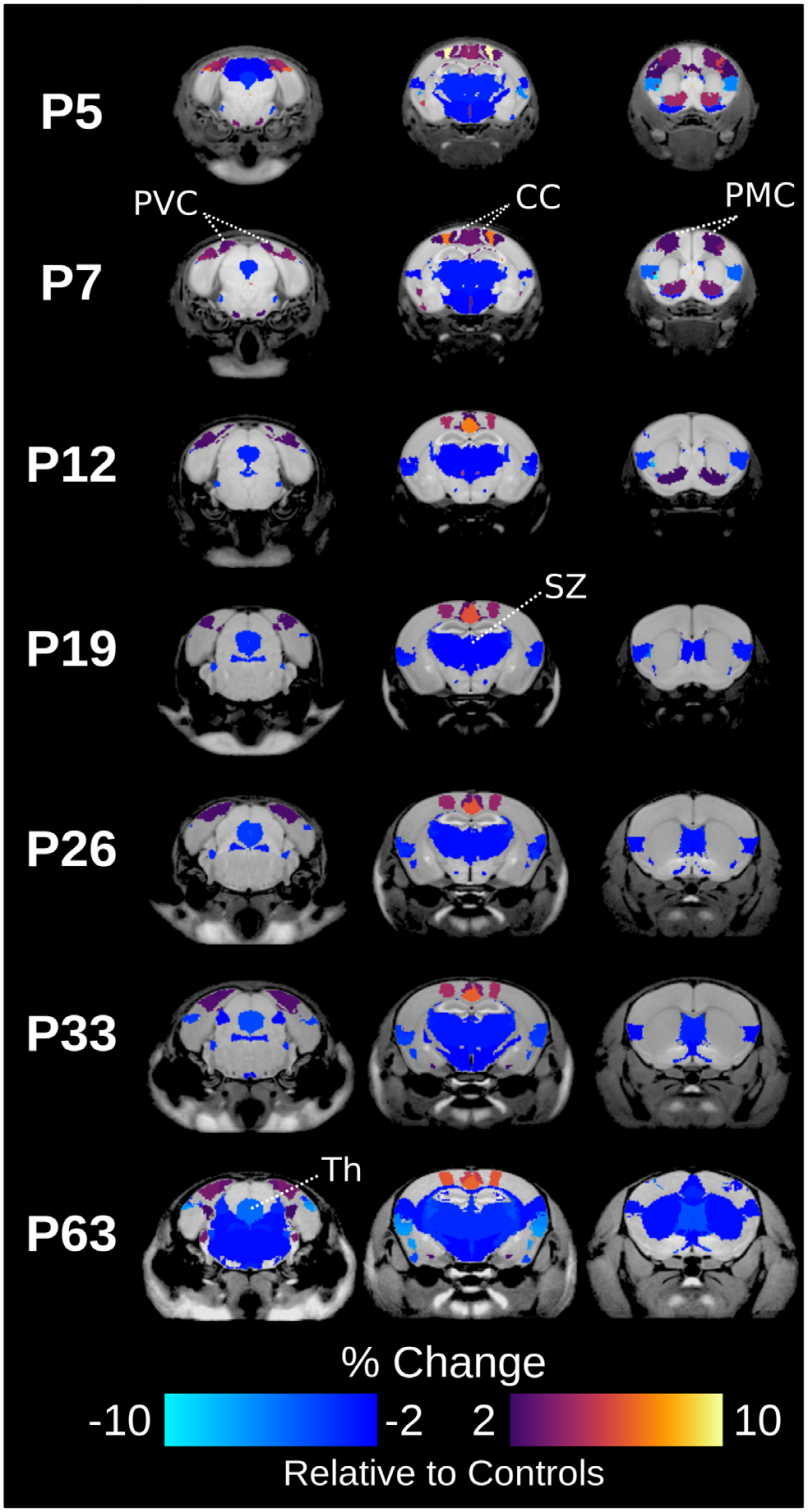
Brain map indicating areas with volume changes larger than 2% in the postterm pregnancy group compared to controls across timepoints. Primary motor cortex: PMC, Primary visual cortex: PVC, Cingulate cortical area 24.1: CC, Thalamus: Th, Subependymale zone rhinocele: SZ.

In addition to focal changes, there were widespread diffuse volume changes with visible patterns as seen in Figure 5 that did not meet the threshold for significance. The postterm group had widespread structural differences with volumes that were 2% smaller relative to controls. The most widespread differences between postterm and control were at P63 (Supplementary Table 1). At P5 and P7, 24 structures within cortical regions were 2% larger in the postterm pregnancy relative to controls (Supplementary Table 2). Subcortical areas that were 2% smaller in postterm group compared to controls across all timepoints include the thalamus, claustrum, and stria terminalis (Supplementary Table 3). Hierarchical analysis of neuroanatomy did not reveal any additional significant differences.

## 4 Discussion

Prolonging pregnancy in mice for 24 h resulted in placental insufficiency indicated by high FPWR, late-onset fetal growth restriction, and altered postnatal brain development. While fetuses born postterm did not differ in their whole-brain volume developmental trajectories, they had significantly smaller absolute and relative volumes of the perirhinal and ectorhinal cortices compared to controls. Brain volume differences became more apparent with increasing postnatal age, with the largest changes observed in adulthood.

Postterm pregnancy led to a significant reduction in perirhinal cortex volume, an area in the medial temporal lobe crucial for recognition memory (Buckley, 2005). A similar decrease was observed in the ectorhinal cortex, a neighboring structure located superior to the perirhinal cortex. Both regions share comparable functions, acting as integrative hubs for inputs from diverse brain areas (Nishio et al., 2018). Rodent studies show that perirhinal cortex lesions impair object recognition and the processing of complex stimuli (Eacott et al., 2001; Kealy & Commins, 2011; Myhrer, 2000). Such memory deficits, involving multiple cortical regions, have also been reported in human populations with FGR (Geva et al., 2006).

In the present study, the effect of postterm pregnancy on neuroanatomy was largest in adulthood. This is consistent with a study in macaques where a neonatal lesion in the perirhinal cortex resulted in impairments in memory recognition that became more severe with age (Zeamer et al., 2015). This functional sparing observed at the neonatal stage was attributed to the heightened plasticity of the immature brain. The findings of Zeamer et al. and the current study, reflect the developmental origins of health and disease hypothesis (DOHaD) (Barker, 2004), where periods of development can overlap with windows of increased vulnerability to illness. Future studies could use a battery of behavioral tests to assess the long-term impact of late-onset fetal growth restriction on brain function. In placental insufficiency, the diminished supply of oxygen and nutrients to the fetus at a critical developmental period triggers compensatory signaling pathways that sustain survival and organogenesis (Jensen et al., 1999). However, this adaptation seems to occur at compromised resilience to disease, increasing the fetus’s susceptibility to illnesses later in life (Nobile et al., 2022). Nevertheless, the increased plasticity during this period may enable a heightened responsiveness to treatments. Consequently, timely diagnosis and implementation of early interventions during this window may significantly improve long-term outcomes.

In clinical studies, depending on the gestational age of placental insufficiency onset and delivery date, heterogeneous patterns of cerebral injury occur (S. L. Miller et al., 2016). For example, there may be a higher vulnerability of grey matter in FGR neonates (Padilla et al., 2014), particularly in the amygdala, basal ganglia, thalamus and insula, left occipital, and parietal lobes. In contrast, white matter had increased fractional anisotropy and axial diffusivity on MRI (Padilla et al., 2014). Decreased relative cortical grey matter volumes have also been seen at term and at birth in FGR, with ensuing functional deficits including reduced attention-interaction capacity (Tolsa et al., 2004).

In the current study, non-significant patterns of altered brain development were seen in cortical and subcortical areas. Offspring from postterm pregnancies showed a trend of larger grey matter cortical structures between P5-7. Similarly, clinical studies show fetuses with FGR had accelerated cortical development despite decreased total brain volumes (Businelli et al., 2015). Levels of cortical folding were also found sufficient (measured by an increased cortical sulcation index proportional to the cortical surface area) in 12-month FGR neonates (Dubois et al., 2008), suggesting mechanisms that prioritize cortical development are active. In mice, this may manifest in the trending volume increase in grey matter cortical areas seen here. Future mouse studies using diffusion tensor imaging to examine white matter microstructure may provide a more sensitive measure to separate these trends (Wu et al., 2013). Additional behavioral testing in mice can link structural deficits with functional outcomes. Although whole-brain volumes were maintained in the postterm group, the subependymale zone, a subcortical area responsible for adult neurogenesis (Kazanis, 2009), was 2% smaller in the postterm group compared to controls across all timepoints. Reduced neurogenesis may partly account for the growing reduction in volumes of the perirhinal and ectorhinal cortex.

Brain maturation differs between mice and humans depending on the process, though cortical formation via neuronal migration is largely complete by term in both (Pressler & Auvin, 2013). In mice, fetal hypoxia at E19.5 disrupts processes peaking at that stage, such as synaptogenesis and regressive events that refine connections and correct neuronal migration errors (Stiles & Jernigan, 2010; Zeiss, 2021). These disruptions may explain the enlarged cortical areas in the postterm group at P5; however, most returned to control volumes by P19, suggesting compensatory pruning of redundant connections.

Other preclinical studies have reported non-uniform changes to the offspring brain following an adverse fetal environment. For example, in a mouse model of maternal malnutrition, the structural connectivity in the brain of the offspring was found to be heterogenous, with brain regions with greater connections found to be more affected (Barbeito-Andrés et al., 2018). Systemic hypoxia during early mouse postnatal brain development resulted in changes in neurogenesis that are dependent on maturational stage and brain region (Schneider et al., 2012). While the potential mechanisms to explain the increased susceptibility of the perirhinal and ectorhinal cortices in this study are unknown, they may be related to structural connectivity or to the timing of the development of these regions of the cortex.

Brain sparing is a fetal compensatory mechanism that increases blood flow to the brain in response to cerebral hypoxia. It has been suggested that frontal lobes are preferentially perfused as both an early and sustained response to chronic hypoxia (Dubiel et al., 2002). Although brain sparing was not directly measured in this study, at P5, under the assumption that volume reflects perfusion, all eight frontal regions showed variable volume trends, with only three sustaining a 2% enlargement. In the same late-onset FGR mouse model, Smolina et al. (2023) reported increased brain perfusion, frontal lobe sparing, and poorer outcomes in delayed or weaker brain sparers (stillbirth, cerebral hypoxia). In humans, brain-sparing FGR fetuses show preserved development and higher IQ at age 3, but this benefit fades by age 5, suggesting protection is limited to early stages development (Scherjon et al., 1998, 2000). Future studies combining *in utero* brain sparing with postnatal structural analysis in this model may help establish a predictive role for cerebral perfusion patterns in FGR.

Despite the known susceptibility of male offspring to hypoxic injury (Hill & Fitch, 2012), we did not find the impact of postterm pregnancy to be dependent on biological sex. Yet, sex differences were observed in the medial amygdala, a well-defined sex-specific area in mice (Qiu et al., 2018). The medial preoptic nucleus of the hypothalamus is also known to be larger in C57BL/6J strain male mice (Qiu et al., 2018); however in the current study, the medial preoptic nucleus of the postterm was 2% smaller than control across all ages independent of sex. Hence, it is unlikely that this area has a sex-specific vulnerability to late-onset FGR. Rather, subtle strain differences between the CD-1 strain used in the current study may account for such differences. In this study, mice were not given unique identifiers at birth thus preventing the assessment of sex effects on fetal weight and FPWR.

The current mouse model uses progesterone injections late in gestation to induce FGR, mimicking third-trimester onset FGR in humans, along with cross-fostering to study postnatal brain development. This approach provides a unique advantage by enabling the tracking of neurodevelopment from the prenatal period to adulthood. However, as a key steroid hormone in regulating brain development, the use of progesterone to prolong gestation may also influence brain outcomes. Clinical trials have explored progesterone’s neuroprotective effects in patients with congenital heart disease (Gaynor et al., 2024) and traumatic brain injury (Skolnick et al., 2014), while animal studies have demonstrated roles for progesterone’s metabolites in mediating hypoxia-induced cell death (Hirst et al., 2007), remyelination (Hussain et al., 2011) and reducing inflammation and oxidative stress in the fetal brain (Ginsberg et al., 2018). These neuroprotective effects may partially explain the mild brain abnormalities observed in our results.

Another limitation of this model is the lack of clinical literature on postterm pregnancies for comparison, owing to the extremely high risk of perinatal mortality (Wennerholm et al., 2019). While FGR babies are often delivered at premature developmental stage, this model delivers at a mature stage of organ development. Therefore, it is important to carefully consider whether the brain injury seen in this study stems from placental insufficiency and FGR, timing of birth or a combination of both factors.

Late-onset FGR is often missed clinically, and only recognized following birth, commonly with a small placenta that may show evidence of underlying disease such as fetal vascular malperfusion (Gaffney et al., 1994; Jöud et al., 2020; Spinillo et al., 2023). Such circumstances also place the newborn at a greater risk of cerebral palsy which has perinatal origins and is commonly diagnosed as a neonate (Redline & Ravishankar, 2018; Sandran et al., 2024; Strand et al., 2016). In this study, late-onset FGR is modeled through the prolongation of gestation which is also associated with a higher incidence of neonatal death, poor school performance, and a higher incidence of metabolic syndrome (Figueras et al., 2018). Although placental analysis was not performed in this study, Smolina et al. (2024) reported reductions in placenta junctional zones with increasing gestation in the same mouse model. Mechanisms driving this reduction included glycogen deposition, increased apoptosis, karyorrhexis, labyrinth atrophy, laminar necrosis, and calcification (Smolina et al., 2024). Despite its limitations, this model advances our understanding of underlying pathophysiology in placental insufficiency induced late-onset FGR.

While administration of manganese chloride in this study improves the contrast of the MRI images (particularly in the olfactory bulb, cerebellum and hippocampus), there are concerns about cellular toxicity at high doses (Silva et al., 2004). In mouse models of brain disease, abnormal rates of Mn2+ uptake have been reported, potentially confounding the observed differences in neuroanatomy between groups (Silva & Bock, 2008). Qualitatively, we did not observe any differences in the degree of enhancement caused by Mn2+ between the postterm and control groups, suggesting the repeated exposure to manganese chloride in this study is not impacting the neuroanatomy at the macroscopic level.

In this study, late-onset FGR from placental insufficiency led to reduced volumes in the perirhinal and ectorhinal cortices. The early developmental insult resulted in more pronounced structural reductions by adulthood, highlighting how early-life exposures and experiences can shape long-term outcomes. Future work can exploit developmental plasticity to mitigate adverse outcomes and improve long-term brain outcomes in FGR.

## Supplementary Material

Supplementary Material

## Data Availability

The data used in the current study are available from the corresponding author on reasonable request.
