# Supplementary material for: Altered adult brain morphology in a mouse model of late-onset fetal growth restriction
Source: Imaging Neurosci (Camb). 2025 Oct 21;3:IMAG.a.946. doi: 10.1162/IMAG.a.946 (PMC12541470; doi:10.1162/IMAG.a.946)
Supplement: Supplementary Material [file IMAG.a.946_supp.pdf]

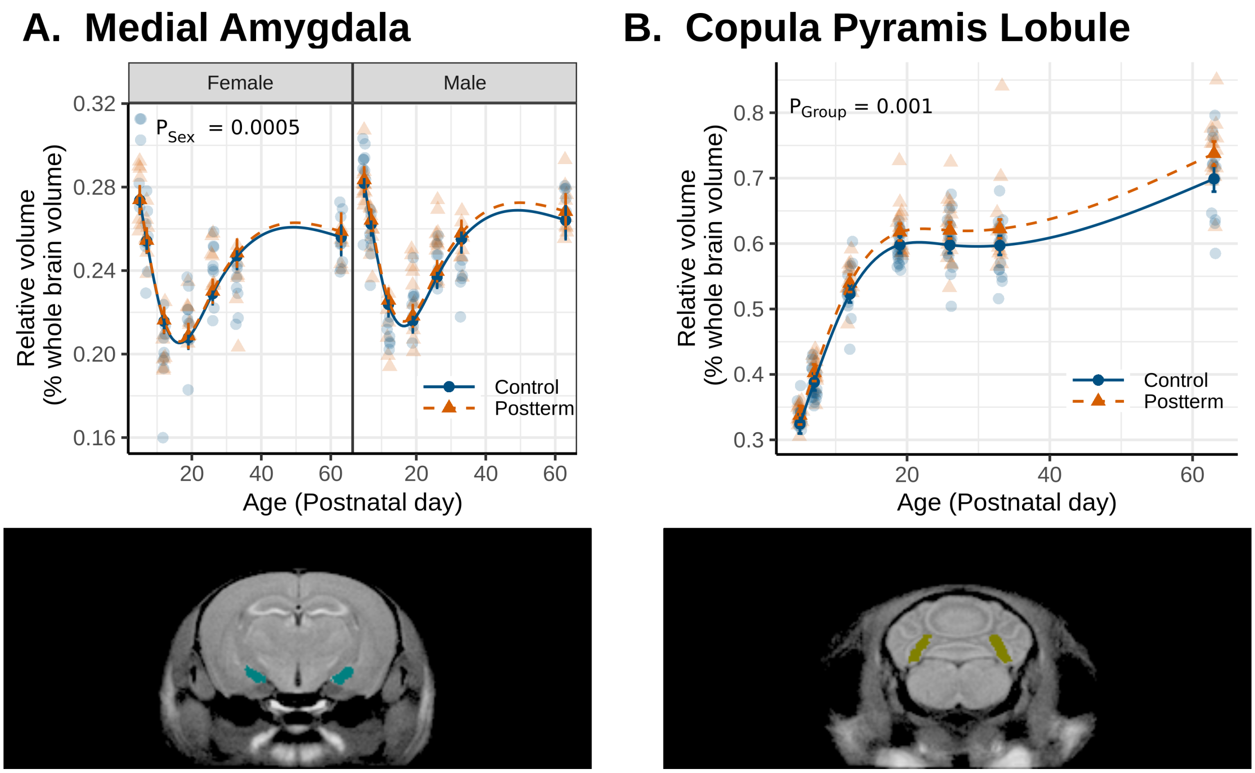

Supplementary Figure 1. A. Relative volume of the medial amygdala shows a significant effect of sex. **B**. Relative volumes of the copula pyramis lobule show a significant group effect.

Supplementary Methods
The following are the linear models used in the paper expressed here in a simplified notation based on the R statistical language.

The linear model for fitting the fetal weights was defined as:
Fetal weight ~ Group + Randon effect of dam

The linear model for fitting the placental weights was defined as:
Placental weight ~ Group + Randon effect of dam

The linear model for fitting the fetal placental weight ratios was defined as:
Fetal placental weight ratio ~ Group + Randon effect of dam

The linear model for fitting the maternal weights was defined as:
*Maternal weight ~ Group * Gestational age + Randon effect of dam*

The linear model for assessing if maternal weight differed between normal and post-gestational intervals was defined as:
Maternal weight ~ Gestational age + Interval + Randon effect of dam

The linear model for fitting postnatal body weight was defined as:
*Weight ~ Group + Age * Sex + Randon effect of litter*

The model for fitting neuroanatomical data was defined as:
*Structure ~ Non-linear effect of age + Group * Sex + Linear difference between groups with age + Randon effect of individual mice*

Supplementary Table 1. Neuroanatomical percent volume difference of the postterm group compared to the control group.

Age (Postnatal Day) / Structure	Percent difference (%)						
	P63	P33	P26	P19	P12	P7	P5
amygdala	0.41	0.55	0.56	0.58	0.68	0.95	1.15
anterior_commissure_pars_anterior	-2.16	-1.64	-1.51	-1.39	-1.39	-1.56	-1.69
anterior_commissure_pars_posterior	-1.39	-0.47	-0.19	0.10	0.46	0.89	1.16
basal_forebrain	1.48	0.02	-0.33	-0.69	-1.22	-2.07	-2.70
bed_nucleus_of_stria_terminalis	-4.30	-3.14	-2.60	-2.10	-1.93	-2.24	-2.53
cerebellar_peduncle_inferior	-0.88	0.39	0.82	1.49	2.83	4.99	6.63
cerebellar_peduncle_middle	-4.18	-3.20	-3.23	-3.50	-3.86	-3.86	-3.79
cerebellar_peduncle_superior	-3.15	-2.60	-2.65	-2.89	-3.47	-4.32	-4.91
cerebral_aqueduct	-5.57	-4.25	-4.54	-5.38	-5.74	-4.63	-4.11
cerebral_peduncle	-2.51	-1.72	-1.56	-1.43	-1.36	-1.37	-1.39
colliculus_inferior	-3.24	-2.10	-1.74	-1.39	-1.16	-1.12	-1.13
colliculus_superior	-1.47	-1.58	-1.51	-1.44	-1.55	-1.92	-2.18
corpus_callosum	-2.53	-0.97	-0.59	-0.19	0.26	0.72	0.96
corticospinal_tract_pyramids	-3.23	-2.01	-1.12	-0.48	-0.09	0.28	0.60
cuneate_nucleus	5.48	4.89	4.64	4.39	4.40	4.76	4.99
facial_nerve_cranial_nerve_7	-1.43	-1.16	-1.18	-1.31	-1.68	-2.49	-3.80
fasciculus_retroflexus	0.98	-0.62	-1.31	-2.91	-6.80	-9.40	-10.04
fimbria	-3.92	-2.67	-2.40	-2.15	-1.98	-1.95	-1.96
fornix	-2.61	-2.47	-2.36	-2.24	-2.26	-2.46	-2.59
fourth_ventricle	0.41	0.90	1.02	1.15	1.41	1.81	2.05
fundus_of_striatum	-3.17	-1.77	-1.23	-0.53	0.39	1.08	1.35
globus_pallidus	-3.27	-2.13	-1.89	-1.68	-1.66	-1.98	-2.27
habenular_commissure	3.78	2.45	2.32	2.32	2.25	1.85	1.55
hypothalamus	-2.15	-2.04	-1.90	-1.76	-1.83	-2.19	-2.45
inferior_olivary_complex	-50.15	9.68	5.43	4.25	4.99	8.09	10.85
internal_capsule	-3.54	-1.79	-1.46	-1.13	-0.72	-0.22	0.13
interpedunclar_nucleus	-2.58	-1.70	-1.29	-0.93	-0.74	-0.78	-0.84
lateral_olfactory_tract	2.14	0.36	-0.10	-0.62	-1.50	-3.47	-5.78
lateral_septum	-4.49	-3.00	-2.56	-2.13	-1.84	-1.78	-1.77
lateral_ventricle	-4.82	-3.60	-3.36	-3.17	-3.04	-3.00	-2.99
mammillary_bodies	-1.46	-1.39	-1.20	-1.01	-0.97	-1.16	-1.31
mammilothalamic_tract	-3.44	-0.29	0.48	1.79	4.95	11.10	16.84
medial_lemniscus_medial_longitudinal_fasciculus	-2.76	-1.22	-0.75	-0.31	0.13	0.75	1.33
medial_septum	-4.33	-2.89	-2.33	-1.77	-1.41	-1.31	-1.28

medulla	-1.33	-0.52	-0.29	-0.05	0.22	0.57	0.81
midbrain	-2.54	-1.80	-1.61	-1.43	-1.36	-1.48	-1.57
nucleus_accumbens	0.70	1.30	1.43	1.60	2.08	3.29	4.38
olfactory_peduncle	2.61	2.47	2.30	2.13	2.28	3.00	3.57
olfactory_tubercle	1.61	0.41	0.10	-0.28	-0.85	-1.70	-2.33
optic_tract	-0.59	-0.97	-1.10	-1.28	-1.65	-2.27	-2.68
periaqueductal_grey	-5.45	-4.32	-3.78	-3.24	-3.06	-3.32	-3.52
pons	-2.38	-1.70	-1.56	-1.45	-1.46	-1.68	-1.85
pontine_nucleus	-2.20	-2.18	-1.98	-1.76	-1.78	-2.16	-2.46
posterior_commissure	-6.57	-5.29	-5.26	-5.42	-5.90	-6.59	-6.98
pre_para_subiculum	2.09	1.78	1.53	1.30	1.30	1.62	1.89
stria_medullaris	-0.42	-0.89	-1.03	-1.21	-1.56	-2.13	-2.51
stria_terminalis	-4.28	-2.85	-2.67	-2.58	-2.72	-3.19	-3.57
striatum	-2.39	-1.50	-1.27	-1.05	-0.92	-0.96	-1.01
subependymale_zone_rhinocele	-3.79	-4.85	-5.17	-5.57	-6.15	-6.77	-7.06
superior_olivary_complex	-1.54	-0.12	0.17	0.53	1.18	2.31	3.24
thalamus	-3.36	-2.64	-2.37	-2.12	-2.18	-2.74	-3.22
third_ventricle	-3.72	-1.03	-0.22	0.66	1.67	2.60	3.05
ventral_tegmental_decussation	3.54	2.59	2.82	3.57	5.05	6.07	6.45
lobules_1_2_lingula_and_central_lobule_ventral	-5.25	-3.89	-3.38	-2.88	-2.85	-3.88	-5.15
lobule_3_central_lobule_dorsal	-0.54	-0.38	-0.35	-0.34	-0.38	-0.58	-0.83
lobules_4_5_culmen_ventral_and_dorsal	1.84	1.00	0.75	0.52	0.36	0.27	0.19
lobule_6_declive	1.63	1.17	1.05	0.96	1.06	1.71	2.70
lobule_7_tuber_or_folium	-0.76	-1.16	-1.33	-1.63	-2.49	-5.22	-9.59
lobule_8_pyramis	1.12	0.21	0.00	-0.18	-0.46	-1.39	-3.71
lobule_9_uvula	4.55	1.74	0.99	0.32	-0.38	-1.78	-3.92
lobule_10_nodulus	1.90	1.55	1.27	1.02	1.02	1.50	2.12
anterior_lobule_lobules_4_5	-2.51	-1.96	-1.79	-1.63	-1.76	-2.73	-4.27
simple_lobule_lobule_6	-0.75	0.27	0.53	0.82	1.43	3.09	5.32
crus_1_ansiform_lobule_lobule_6	-2.40	-1.79	-1.66	-1.58	-1.88	-3.36	-5.85
crus_2_ansiform_lobule_lobule_7	-4.27	-3.63	-3.49	-3.48	-4.45	-8.80	-16.89
paramedian_lobule_lobule_7	1.10	0.24	0.03	-0.19	-0.52	-1.48	-3.32
copula_pyramis_lobule_8	3.60	2.19	1.71	1.22	0.84	0.56	0.25
flocculus_FL	3.34	1.77	1.38	1.01	0.76	0.65	0.59
paraflocculus_PFL	-2.70	-1.69	-1.31	-0.99	-0.96	-1.65	-3.34
trunk_of_arbor_vita	0.42	-0.48	-0.75	-1.09	-1.69	-2.60	-3.24
lobule_1_2_white_matter	4.32	0.77	0.50	-0.64	6.34	-7.90	-4.19
lobule_3_white_matter	1.28	0.70	0.75	1.08	1.93	1.39	NA

trunk_of_lobules_1_3_white_matter	-3.70	-2.23	-2.63	-4.58	-10.72	-6.75	-4.98
lobules_4_5_white_matter	3.78	2.84	3.18	4.54	9.92	35.01	-1267.39
lobules_6_7_white_matter	-1.05	0.16	0.50	0.94	1.91	5.07	11.77
lobule_8_white_matter	-4.23	-1.00	0.63	2.25	4.85	12.57	26.35
trunk_of_lobules_6_8_white_matter	0.68	1.71	2.30	3.54	6.49	11.60	15.92
lobule_9_white_matter	-1.01	13.51	9.04	6.13	6.41	10.18	13.96
lobule_10_white_matter	1.00	-0.04	-0.64	-1.65	-3.52	-5.80	-7.14
anterior_lobule_white_matter	-7.02	0.96	3.83	12.60	110.01	-172.78	-117.06
simple_lobule_white_matter	-3.57	1.05	3.21	7.27	21.76	-1090.84	-60.85
crus_1_white_matter	2.53	1.42	1.07	0.59	-0.22	-1.44	-2.38
trunk_of_simple_and_crus_1_white_matter	-2.21	-3.60	-3.76	-3.75	-4.74	-10.40	-26.61
crus_2_white_matter	-12.43	-7.09	-8.52	-17.18	-169.01	-50.56	NA
paramedian_lobule	-5.32	0.81	5.12	313.34	-33.37	NA	NA
trunk_of_crus_2_and_paramedian_white_matter	1.58	1.75	2.05	2.77	4.63	8.40	NA
copula_white_matter	-0.10	-1.41	-2.05	-3.20	-5.86	-11.32	-16.93
paraflocculus_white_matter	-1.28	0.31	0.93	2.05	5.27	20.53	299.56
flocculus_white_matter	-0.43	1.30	2.20	4.42	96.15	-6.69	-4.77
dentate_nucleus	2.88	1.91	1.72	1.54	1.41	1.37	NA
nucleus_interpositus	3.51	1.56	1.12	0.67	0.12	-0.50	-0.89
fastigial_nucleus	3.24	1.13	0.60	0.03	-0.67	-1.41	-1.82
cingulate_cortex_area_24a	-3.19	-1.07	-0.58	-0.13	0.39	1.34	2.45
cingulate_cortex_area_24a.1	6.55	6.37	6.16	6.06	7.18	10.76	14.07
cingulate_cortex_area_24b	-2.20	-0.85	-0.52	-0.21	0.11	0.61	1.13
cingulate_cortex_area_24b.1	5.32	3.22	2.89	2.64	2.54	2.73	3.02
cingulate_cortex_area_25	-0.38	-0.13	-0.05	0.02	0.09	0.21	0.29
cingulate_cortex_area_29a	2.66	1.66	1.28	0.98	0.93	1.27	1.63
cingulate_cortex_area_29b	4.98	2.89	2.64	2.52	2.88	4.22	5.61
cingulate_cortex_area_29c	0.39	1.48	1.59	1.67	2.08	3.15	4.04
cingulate_cortex_area_30	1.88	1.76	1.67	1.61	1.86	2.72	3.51
cingulate_cortex_area_32	-1.69	-0.92	-0.76	-0.61	-0.55	-0.64	-0.76
amygdalopiriform_transition_area	-0.25	1.22	1.64	2.13	3.13	5.24	7.13
primary_auditory_cortex	-3.14	-0.97	-0.39	0.14	0.78	1.83	2.78
secondary_auditory_cortex_dorsal_area	-1.45	-0.82	-0.69	-0.58	-0.56	-0.72	-0.94
secondary_auditory_cortex_ventral_area	-3.39	-1.35	-0.78	-0.29	0.16	0.74	1.22
caudomedial_entorhinal_cortex	0.38	0.49	0.48	0.47	0.51	0.66	0.76
cingulum	0.66	1.41	1.70	2.09	2.58	2.93	3.08
claustrum	-3.94	-3.94	-4.40	-5.48	-7.51	-9.73	-10.97
cortex_amygdala_transition_zones	-1.11	-2.01	-2.20	-2.37	-2.71	-3.21	-3.49

claustrum_dorsal_part	-3.24	-1.96	-1.96	-2.28	-2.53	-1.50	-0.96
dorsal_nucleus_of_the_endopiriform	-0.78	-0.74	-0.71	-0.70	-0.78	-1.05	-1.27
dorsal_intermediate_entorhinal_cortex	-0.26	0.46	0.64	0.81	1.14	1.84	2.41
dorsolateral_entorhinal_cortex	1.03	1.31	1.25	1.17	1.31	1.81	2.23
dorsolateral_orbital_cortex	-2.13	-0.71	-0.52	-0.33	-0.04	0.45	0.92
dorsal_tenia_tecta	6.45	5.17	5.08	5.20	5.93	7.62	8.96
ectorhinal_cortex	-6.30	-3.67	-3.05	-2.45	-2.06	-2.12	-2.33
frontal_cortex_area_3	-5.57	-1.93	-1.01	-0.05	1.20	3.43	5.65
frontal_association_cortex	0.26	0.04	-0.01	-0.06	-0.13	-0.24	-0.32
intermediate_nucleus_of_the_endopiriform_claustrum	-1.56	-1.97	-2.16	-2.44	-3.17	-4.85	-6.45
insular_region_not_subdivided	-1.93	-2.08	-2.26	-2.60	-3.43	-5.05	-6.34
lateral_orbital_cortex	-0.74	0.18	0.40	0.64	1.10	2.18	3.38
lateral_parietal_association_cortex	-1.13	-0.07	0.22	0.55	1.09	2.13	3.07
primary_motor_cortex	0.42	0.99	1.07	1.15	1.47	2.32	3.09
secondary_motor_cortex	1.66	1.15	1.06	1.01	1.10	1.46	1.79
medial_entorhinal_cortex	0.37	0.46	0.46	0.46	0.56	0.88	1.20
medial_orbital_cortex	-3.00	-2.03	-1.93	-1.91	-2.07	-2.46	-2.74
medial_parietal_association_cortex	0.26	1.63	1.80	1.90	2.18	2.70	3.02
piriform_cortex	0.94	0.79	0.74	0.70	0.75	0.95	1.11
posterolateral_cortical_amygdaloid_area	-1.57	-0.74	-0.53	-0.31	-0.05	0.41	1.01
posteromedial_cortical_amygdaloid_area	2.15	2.07	1.79	1.51	1.62	2.53	3.64
perirhinal_cortex	-7.00	-3.89	-3.38	-2.91	-2.42	-1.78	-1.13
parietal_cortex_posterior_area_rostral_part	1.90	-1.32	-2.25	-3.49	-5.47	-8.02	-9.56
rostral_amygdalopiriform_area	-4.20	-1.35	-0.68	0.14	1.45	3.83	6.07
primary_somatosensory_cortex	-2.05	-0.83	-0.52	-0.17	0.33	1.21	2.08
primary_somatosensory_cortex_barrel_field	-0.19	-0.07	-0.04	-0.02	0.02	0.06	0.09
primary_somatosensory_cortex_dysgranular_zone	1.28	1.59	1.76	2.10	3.28	9.02	39.94
primary_somatosensory_cortex_forelimb_region	1.31	1.34	1.30	1.28	1.51	2.25	2.92
primary_somatosensory_cortex_hindlimb_region	6.15	4.47	4.10	3.85	4.43	7.03	10.15
primary_somatosensory_cortex_jaw_region	-0.48	-0.66	-0.84	-1.31	-2.61	-4.54	-5.91
primary_somatosensory_cortex_shoulder_region	13.15	5.87	5.63	6.17	9.33	38.88	-46.17
primary_somatosensory_cortex_trunk_region	1.44	0.68	0.58	0.51	0.38	0.19	0.07
primary_somatosensory_cortex_upper_lip_region	-1.91	-0.88	-0.63	-0.36	-0.03	0.47	0.96
secondary_somatosensory_cortex	-2.44	-1.38	-1.16	-0.97	-0.87	-0.97	-1.13
temporal_association_area	-3.28	-1.94	-1.65	-1.41	-1.36	-1.75	-2.28
primary_visual_cortex	3.36	2.45	2.30	2.22	2.49	3.49	4.46
primary_visual_cortex_binocular_area	3.57	2.15	1.70	1.30	1.12	1.32	1.59
primary_visual_cortex_monocular_area	4.48	2.18	1.69	1.21	0.79	0.43	0.20

secondary_visual_cortex_lateral_area	1.21	1.35	1.42	1.58	2.16	4.02	6.59
secondary_visual_cortex_mediolateral_area	2.77	2.03	1.89	1.79	1.91	2.38	2.73
secondary_visual_cortex_mediomedial_area	2.79	2.39	2.16	1.96	2.10	2.87	3.56
claustrum_ventral_part	-1.54	-0.88	-0.82	-0.82	-0.79	-0.60	-0.47
ventral_nucleus_of_the_endopiriform_claustrum	-6.99	-3.77	-2.84	-1.60	0.57	3.49	5.32
ventral_intermediate_entorhinal_cortex	-1.67	-1.57	-1.44	-1.31	-1.39	-1.85	-2.26
ventral_orbital_cortex	-1.58	-1.18	-1.04	-0.90	-0.90	-1.22	-1.57
ventral_tenia_tecta	1.49	-0.34	-0.87	-1.56	-2.48	-3.35	-3.75
CA10r	-0.65	-0.13	0.01	0.17	0.39	0.74	1.01
LMol	0.00	0.22	0.27	0.32	0.44	0.77	1.11
CA1Rad	0.40	0.38	0.36	0.34	0.39	0.59	0.78
CA2Py	-1.31	-0.23	0.10	0.54	1.33	2.75	3.98
CA20r	-0.31	0.18	0.33	0.53	0.92	1.84	2.85
CA2Rad	-2.08	-1.19	-0.92	-0.65	-0.44	-0.29	-0.15
CA3Py_Inner	-0.27	-0.62	-0.74	-0.93	-1.46	-3.22	-6.39
CA3Py_Outer	-1.66	-0.75	-0.51	-0.26	0.04	0.44	0.75
CA30r	-0.91	-0.56	-0.49	-0.42	-0.37	-0.35	-0.34
CA3Rad	-1.97	-1.00	-0.70	-0.42	-0.16	0.12	0.35
SLu	-2.41	-1.17	-0.87	-0.35	1.41	6.24	11.87
MoDG	-0.34	0.03	0.12	0.22	0.36	0.61	0.80
GrDG	-1.86	-0.84	-0.54	-0.23	0.15	0.82	1.62
PoDG	0.10	0.21	0.24	0.28	0.41	0.78	1.28
CA1Py	0.31	0.52	0.66	0.91	1.52	2.72	3.90
olfactory_bulb_glomerular_layer	3.84	1.54	1.01	0.34	-0.83	-3.26	-6.10
olfactory_bulb_external_plexiform_layer	0.50	-0.47	-0.74	-1.06	-1.67	-2.89	-3.97
olfactory_bulb_mitral_cell_layer	2.75	1.46	1.27	1.09	0.43	-2.26	-7.31
olfactory_bulb_internal_plexiform_layer	2.87	1.11	0.68	0.00	-1.89	-6.74	-13.11
olfactory_bulb_granule_cell_layer	1.86	0.76	0.44	0.12	-0.24	-0.72	-1.09
accessory_olfactory_bulb_glomerular_ external_plexiform_and_mitral_cell_layer	1.39	1.53	1.58	1.68	2.18	3.71	5.41
accessory_olfactory_bulb_granule_cell_layer	5.80	4.76	4.13	3.56	3.62	4.61	5.43
anterior_olfactory_nucleus	3.80	2.96	2.56	2.20	2.26	3.10	3.93
subiculum	1.23	0.47	0.26	0.06	-0.13	-0.40	-0.63
medial_amygdala	0.96	0.93	0.96	1.02	1.17	1.41	1.56
medial_preoptic_nucleus	-11.99	-10.36	-6.50	-4.16	-3.71	-5.19	-6.95

Supplementary Table 2. Cortical structures in which postterm pregnancy increased volume by greater than 2% at P5 and P7.

**Structure**	**Cortical Region**
Amygdalopiriform transition area	Piriform cortex area
CA1Py	Hippocampal region
CA2Py	Hippocampal region
Cingulate cortex: area 24a'	Cingulate region
Cingulate cortex: area 24b'	Cingulate region
Cingulate cortex: area 29b	Cingulate region
Cingulate cortex: area 29c	Cingulate region
Cingulate cortex: area 30	Cingulate region
Dorsal tenia tecta	Entorhinal cortex
Frontal cortex: area 3	Frontal region
Lateral orbital cortex	Frontal region
Lateral parietal association cortex	Parietal region
Medial parietal association cortex	Parietal region
Posteromedial cortical amygdaloid area	Piriform cortex area
Primary motor cortex	Frontal region
Primary somatosensory cortex: dysgranular zone	Parietal region
Primary somatosensory cortex: forelimb region	Parietal region
Primary somatosensory cortex: hindlimb region	Parietal region
Primary visual cortex	Occipital region
Rostral amygdalopiriform area	Piriform cortex area
Secondary visual cortex: lateral area	Occipital region
Secondary visual cortex: mediolateral area	Occipital region
Secondary visual cortex: mediomedial area	Occipital region
SLu	Hippocampal region

Postterm pregnancy pups had increased volumes greater than 2% compared to controls in the listed structures between P5-P7. The corresponding cortical region for each structure is listed.

Supplementary Table 3. Structures with greater than 2% decrease in subcortical area volumes in all ages across postterm pregnancy.

**Structure**	**Tissue Type**
Cerebral aqueduct	CSF
Claustrum	GM
Fornix	WM
Lateral ventricle	CSF
Medial preoptic nucleus	GM
Periaqueductal grey	GM
Posterior commissure	WM
Stria terminalis	GM
Subependymale zone rhinocele	GM
Thalamus	GM

Pups from postterm pregnancies exhibited a greater than 2% decrease in subcortical area volume, which was maintained across all ages. *Grey matter: GM., white matter: WM, Cerebropsinal fluid: CSF.*
